# Temporal evolution of the risk factors associated with low birth weight rates in Brazilian capitals (1996-2011)

**DOI:** 10.1186/s12963-016-0086-0

**Published:** 2016-05-03

**Authors:** Viviane Costa de Souza Buriol, Vânia Hirakata, Marcelo Zubaran Goldani, Clécio Homrich da Silva

**Affiliations:** Graduate Program in Child and Adolescent Health, School of Medicine, Universidade Federal do Rio Grande do Sul, Rua Ramiro Barcelos, 2400/2° andar. Barrio Santa Cecilia, Porto Alegre, RS 90035-003 Brazil; Child and Adolescent Health Study Centre, Universidade Federal do Rio Grande do Sul, Porto Alegre, Brazil; Hospital de Clínicas de Porto Alegre, Porto Alegre, Brazil; Department of Pediatrics, School of Medicine, Universidade Federal do Rio Grande do Sul, Porto Alegre, Brazil

**Keywords:** Low birth weight, Maternal and child health, Information system

## Abstract

**Objective:**

To analyze the trend of low birth weight (LBW) and its determinants in Brazilian state capitals between 1996 and 2011. We intended to determine which variables are associated with LBW during the period studied.

**Methods:**

This is a cross-sectional study that used data from the National Information System of Live Births from 26 state capitals and Brasilia (the federal capital), divided into five geographical regions. The Average Annual Percentage of Change (AAPC) was used to assess the possible time trend in the low birth weight rates for considering all regions together and each region separately, according to each variable, and the Poisson regression was calculated in order to demonstrate time trends in low birth weight and the impact of variables (age and educational maternal level, antenatal visits, type of delivery, and gestational age) during the period. All variables were analyzed together using the Poisson regression as well.

**Results:**

From the total of 11,200,255 live births used in this study, there was a significant reduction in the number of live births, especially in the more developed regions. The low birth weight rate was 8 %, and it was stable during the period. Considering regional trends, the rate was higher in the Southeast and South regions, and significantly higher in the North, Northeast, and Central West regions. Improvements in maternal education and antenatal care coverage reduced the risk for low birth weight in all regions. Also, there was an increase in caesarean sections in all regions, with a small impact on low birth weight rates.

**Conclusions:**

Improvements in education and health care reduced the risk for low birth weight in all Brazilian regions during the period of study. Trends in low birth weight rates and the associated factors differ from region to region, showing different stages of demographic, epidemiological and developmental transition in Brazil. The present study was approved by the Research Ethics Committee at the Hospital de Clínicas de Porto Alegre (Protocol 120323).

## Background

Newborns under 2,500 grams (g) are considered to have low birth weight (LBW), which is a major factor associated with infant morbidity and mortality [1–4]. As a consequence, the chance of preterm newborns and very small infants surviving is lower [5]. LBW has also been associated with adverse effects on child development throughout life, such as higher predisposition to chronic diseases [6, 7], problems related to affective-motivational aspects [8], cognitive performance difficulties [9], and negative psychological and emotional consequences [10, 11]. The cause of LBW is multifactorial. Duration of pregnancy and intrauterine growth play an important role regarding LBW [12]. Maternal socioeconomic conditions and maternal pregnancy diseases also influence the development of the fetus and the prevalence of LBW [13, 14]. The factors directly impacting the intrauterine fetal growth include: gender of the newborn, size of the newborn, if the newborn is underweight at birth; the mother’s weight gain and caloric intake during pregnancy, if there was smoking or alcohol consumption during pregnancy, and the mother’s weight at delivery. Among the indirect factors (i.e., those with impacts that are expressed by a direct factor) are the age, the socioeconomic status of the mother, and race or ethnic origin [12].

Every year, it is estimated that about 20 million children are born underweight worldwide, and 95.6 % of these births occur in developing countries [15]. In developed countries, LBW affects predominantly preterm newborns, whereas in developing countries most LBW infants are born at term but suffer intrauterine growth restriction [13, 14, 16].

Brazil has experienced an intense demographic and epidemiological transition characterized by change in its age structure, infant mortality reduced rates, decreased fertility rates, and population aging [17]. The access to perinatal technological devices and services have decreased infant mortality rates and increased LBW rates in recent years. Even with this progress, the quality of antenatal care is poorer in more vulnerable social groups [18]. Interestingly, many studies have demonstrated that increased LBW rates in Brazil have a paradoxical distribution since the highest rates are found in regions with higher socioeconomic development [18, 19]. Low birth weight was also associated with multiple births and stillbirths as a secular trend in Brazil [20].

In order to collect data for births in Brazil, the Ministry of Health created and implemented the National Information System of Live Births (SINASC), and since the 1990s have made it possible to understand certain aspects of health regarding newborns and their mothers from an epidemiological perspective. Its implantation was gradual throughout the country but developed at a faster rate in more economically developed cities. In recent years, analysis of information for the year 2000 showed an improvement all over the country, mainly in the North and Northeast regions, known as the less socioeconomically developed regions [21]. Compared to other cities from less developed regions, whose data are more precarious [22], the capitals’ count is more reliable especially in the early years of the registry implementation. However, no prior study assessed the main causes of LBW in Brazil, considering this heterogenic socioeconomic regional distribution.

The objective of the present study was to investigate LBW rate trends in Brazil from 1996 to 2011 and to identify which variables (educational level and maternal age, number of antenatal visits, gestational age, and type of delivery) influenced this trend by analyzing the data from 26 state capitals and Brazil’s capital, Brasilia. The variables that have a close relationship with LBW in the current literature were selected in order to determine which of these variables are associated with LBW during the period studied. Understanding the relationship between these variables and LBW for a period of time could inform potential development of actions that could minimize the occurrence of LBW in Brazil.

## Methods

This cross-sectional study included data analyzed from 26 state capitals and Brasilia (the federal capital) located in five different geographic regions between 1996 and 2011. Brazil is the largest country in South America, with an estimated population of 202,768,562 inhabitants in 2014 [23]. The country is politically and administratively divided into 26 state capitals and the federal capital, Brasilia, and distributed in five geographic regions: Central West, Northeast, North, Southeast, and South. In terms of socioeconomic development, the Southeast and South are the more developed regions, while the North and the Northeast are the less developed ones, and the Central West region resides between these two poles [24].

Data were collected from the database of the Department of Informatics of the Unified Health System (DATASUS), Ministry of Health, using the National Information System of Live Births (SINASC), which contains the Birth Declaration (BD) of all live births. SINASC is one of the most reliable sources of information and is an important tool for the Ministry of Health, since it allows monitoring outcomes in the area of maternal and child health and the development of health policies [22].

The coverage of SINASC went from 97 % in 2010 to 100 % in 2011 in the South, Southeast, and Central West regions [25]. However, estimates of preterm newborn prevalence, obtained through primary studies using a linear regression model based on fractional polynomial equations, were greater than the estimates by SINASC. This difference may be because the gestational age was informed in weekly intervals until 2011, then the data was collected as a continuous variable (in full weeks) according to the information from the BD [26].

From a total of 11,922,266 live births, this study included 11,200,255 single births, with all single newborns weighing 500 g or more. The records were obtained using the SINASC database, and these results only considered the children who were born in the cities where their mothers live. It excluded 440,680 (3.9 % of all) newborns, as mothers who present high-risk pregnancies are sent to hospitals in the capitals due to the lack of resources and facilities in small cities.

The following variables were used: number of live births, maternal age (10–17 years; 18–34 years; ≥35 years), maternal educational level (<8 years; 8–11 years; ≥12 years), number of antenatal visits (none; ≤6 visits; ≥7 visits), gestational age (<37 weeks; ≥37 weeks), type of delivery (vaginal; caesarean section), and birth weight (from 500 to 2,499 g – LBW or ≥2,500 g). Considering the best outcome for birth weight, the reference values for each variable were: maternal age of 18–34 years, number of antenatal visits ≥7 visits, gestational age ≥37 weeks, and vaginal delivery. The reference value for the maternal educational level was 8–11 years, as the two extreme levels can present similar outcomes, according to the inverse care law published by Tudor-Hart in 1971 [27].

A conceptual framework with low birth weight as the outcome was constructed to attend to the conditional regression procedures through three different levels. The first level presented the annual LBW prevalence by region (Table 1). At the second level, LBW determinants were reported (maternal age and education, antenatal care, gestational age, and mode of delivery) (Table 2). Finally, the third level focused on the annual impact (temporal trend) on LBW, with each independent variable analyzed using the Poisson Regression (Table 3).

**Table 1:** Number of live births and rates of low birth weight (<2,500 g) among single newborns in Brazil and its regions according to the 26 state capitals and Brazil’s capital, Brasília, between 1996 and 2011

	North		Northeast		Southeast		South		Central West		Brazil	
Year	No. of NBs^a^	LBW %^b^	No. of NBs^a^	LBW %^b^	No. of NBs^a^	LBW %^b^	No. of NBs^a^	LBW %^b^	No. of NBs^a^	LBW %^b^	No. of NBs^a^	LBW %^b^
1996	96,544	6.6	142,999	7.0	329,838	8.9	55,853	7.4	88,757	7.2	757,125	8.0
1997	97,758	6.7	138,253	7.0	330,113	8.3	55,816	7.7	88,354	6.9	755,641	7.7
1998	97,070	6.9	148,990	7.4	319,849	8.4	54,946	7.9	89,114	7.2	752,307	7.9
1999	101,800	6.5	154,737	7.3	330,555	8.1	56,501	7.8	88,987	6.8	776,043	7.6
2000	92,156	7.1	150,537	7.3	315,964	8.3	55,508	7.9	87,859	6.9	744,342	7.8
2001	95,380	7.0	148,989	7.5	289,539	8.6	50,807	8.1	84,305	7.4	709,674	8.0
2002	95,236	7.5	140,019	7.7	281,203	8.6	49,364	8.0	83,620	7.4	687,278	8.1
2003	94,366	7.5	143,851	7.9	282,071	8.8	46,877	8.6	83,664	7.7	687,830	8.3
2004	91,815	7.7	138,547	8.0	281,380	8.6	47,535	8.5	83,646	7.4	679,068	8.3
2005	92,576	7.4	137,631	7.8	275,483	8.4	46,382	7.9	84,069	7.4	671,783	8.0
2006	94,099	7.4	136,987	7.8	270,500	8.4	46,158	7.9	83,251	7.3	666,535	8.0
2007	92,593	7.4	132,107	7.7	267,389	8.4	45,273	8.0	81,603	7.4	654,832	8.0
2008	93,642	7.4	135,418	7.9	269,555	8.2	47,184	8.1	82,933	7.5	664,285	8.0
2009	93,228	7.6	132,493	7.8	272,156	8.4	46,875	8.1	82,440	7.7	662,763	8.1
2010	93,202	7.5	129,126	7.7	270,975	8.3	47,126	7.9	83,231	7.7	658,532	8.0
2011	95,611	7.6	134,181	7.8	275,449	8.1	47,502	7.8	83,810	8.0	672,217	8.0
Total	1,517,076	7.2	2,244,865	7.6	4,662,019	8.4	799,707	8.0	1,359,643	7.4	11,200,255	8.0
APC (95 % CI)^c^		1(0.4;1.6)^d^		0.7(0.4;1.0)^d^		−0.5(−1.5;0.6)		0.3(−0.2;0.8)		0.7(0.4;1.1)^d^		0.1(−0.7;0.8)

^a^Newborns; ^b^Low Birth Weight; ^c^ Annual Percentage of Change; ^d^ APC significantly different from zero

**Table 2:** Distribution of maternal characteristics in the first and last years of the series and AAPC^a^ of low birth weight among single newborns for each stratum of the variables studied, Brazil and its regions according to the 26 state capitals and Brazil’s capital, Brasília, between 1996 and 2011

Variables	1996	2011	AAPC (95 % CI)	*P*	1996	2011	AAPC (95 % CI)	*P*	1996	2011	AAPC (95 % CI)	*P*
	North				Northeast				Southeast			
*Maternal age (years)*												
10–17 years	14.9	11.3	-	-	11.4	9.0	-	-	8.0	7.0	-	-
≥35 years	4.7	8.6	-	-	6.4	10.9	-	-	9.7	15.3	-	-
18-34	80.3	80.0	-	-	82.2	80.2	-	-	82.2	77.7	-	-
LBW 10–17 years	10.2	10.6	0.1 (−0.9;1.2)	<0.001	9.9	10.7	0.2 (−0.5;0.9)	0.037	11.9	10.9	−0.5 (−1.5;0.4)	0.227
LBW ≥35 years	6.6	8.6	1.8 (1.0;2.6)^a^	<0.001	7.7	9.0	0.6 (0.2;1.1)^a^	<0.001	11.0	9.3	−0.9 (−1.1;−0.6)^a^	<0.001
LBW 18–34	6.0	7.0	1.1 (0.5;1.8)^a^	<0.001	6.6	7.4	0.8 (0.5;1.1)^a^	<0.001	8.3	7.6	−0.4 (−1.2;0.4)	0.001
*Educational level (years)*												
<8 years	64.2	28.3	-	-	62.3	26.2	-	-	48.9	19.5	-	-
≥12	3.0	16.4	-	-	5.5	19.5	-	-	10.4	24.5	-	-
8–11	32.7	55.3	-	-	32.2	54.3	-	-	40.7	56.0	-	-
LBW <8 years	7.1	8.3	1.2 (0.6;1.9)^a^	<0.001	7.7	9.0	1.1 (0.7;1.5)^a^	<0.001	10.0	9.7	−0.2 (−1.1;0.7)	<0.001
LBW ≥12	5.4	7.5	3.2 (1.3;5.0)^a^	<0.001	5.1	7.3	1.3 (0.6;2)^a^	<0.001	5.7	7.2	1.5 (1.0;2.1)^a^	<0.001
LBW 8-11	5.7	7.2	1.6 (1.3;2.0)^a^	<0.001	6.2	7.5	1.4 (0.7;2.2)^a^	<0.001	7.9	8.0	0.1 (−1.3;1.5)	<0.001
*Prenatal care (no. of visits)*												
None	11.1	4.1	-	-	9.7	4.7	-	-	6.6	1.7	-	-
≤6 or	35.5	49.9	-	-	37.5	46.9	-	-	35.1	24.2	-	-
≥7	53.4	46.0	-	-	52.8	48.4	-	-	58.3	74.1	-	-
LBW None	10.1	13.9	2.7 (−0.2;5.6)	<0.001	11.3	13.2	1.5 (−0.5;3.6)	<0.001	16.9	24.2	3.7 (2.7;4.7)^a^	<0.001
LBW ≤6 or	7.5	9.3	1.3 (0.9;1.8)^a^	<0.001	8.4	9.8	1 (0.1;1.8)^a^	<0.001	11.7	14.0	1.4 (0.4;2.3)^a^	<0.001
LBW ≥7	5.4	5.0	−0.4 (−0.8;0.1)	0.002	5.1	5.2	0.4 (−0.2;0.9)	<0.001	6.1	5.8	−0.3 (−0.5;0.0)^a^	<0.001
Gestational age (weeks)												
<37	4.9	10.8	-	-	4.9	11.0	-	-	6.5	8.7	-	-
≥37	95.1	89.2	-	-	95.1	89.0	-	-	93.5	91.3	-	-
LBW <37	45.1	34.9	1.2 (−1.6;4)	<0.001	48.1	39.4	−1.2 (−3.7;1.3)	<0.001	59.3	52.5	−0.1 (−0.6;0.4)	<0.001
LBW ≥37	4.6	4.2	0.1 (−0.7;0.8)	0.937	4.8	3.7	−1.3 (−2.2;0.4)^a^	<0.001	5.2	3.9	−2 (−2.7;1.3)^a^	<0.001
Mode of delivery												
Caesarean section	34.5	51.2	-	-	38.0	60.3	-	-	47.3	53.1	-	-
Vaginal delivery	65.5	48.8	-	-	62.0	39.7	-	-	52.7	46.9	-	-
Caesarean section LBW	5.3	7.4	2.1 (1.4;2.8)^a^	<0.001	5.8	7.1	1.2 (0.7;1.7)^a^	<0.001	8.0	8.4	0.5 (−0.1;1.1)	<0.001
Vaginal delivery LBW	7.3	7.7	0.4 (0.0;0.9)	<0.001	7.7	9.0	0.7 (0.4;1.1)^a^	<0.001	9.6	7.8	−1.2 (−1.5;−0.9)^a^	<0.001
	South				Central West				Brazil			
*Maternal age (years)*												
10–17 years	9.4	6.7	-	-	10.6	7.0	-	-	10.1	8.1	-	-
≥35 years	10.5	15.4	-	-	5.9	12.3	-	-	8.0	13.1	-	-
18-34	80.1	77.9	-	-	83.5	80.6	-	-	81.9	78.9	-	-
LBW 10–17 years	10.6	10.0	−0.3 (−1.1;0.5)	0.246	10.2	10.3	0.6 (0.1;1.0)^a^	0.001	11.0	10.8	−0.1 (−0.8;0.5)^a^	0.008
LBW ≥35 years	9.2	8.8	−0.5 (−1;−0.1)^a^	0.010	8.9	8.6	0.3 (−0.3;0.8)	0.175	9.8	9.1	−0.4 (−0.6;−0.2)	<0.001
LBW 18–34	6.8	7.5	0.5 (0.0;1.1)	<0.001	6.7	7.7	0.9 (0.5;1.2)^a^	<0.001	7.4	7.5	0.1 (−0.4;0.6)	<0.001
*Educational level (years)*												
<8 years	46.2	20.6	-	-	52.6	18.8	-	-	55.4	22.3	-	-
≥12	10.8	33	-	-	7.4	27.0	-	-	7.2	22.9	-	-
8–11	42.9	46.4	-	-	40.1	54.2	-	-	37.4	54.8	-	-
LBW <8 years	8.6	9.5	1 (0.4;1.6)	<0.001	7.9	9.2	0.7 (0.2;1.2)^a^	<0.001	8.4	9.3	0.6 (−1;2.2)	,0.001
LBW ≥	5.2	6.8	1.8 (0.7;2.9)^a^	<0.001	5.7	7.8	1.8 (1.2;2.3)^a^	<0.001	5.6	7.3	1.5 (1.1;1.9)^a^	,0.001
LBW 8-11	6.6	7.8	1.1 (0.1;2.2)^a^	<0.001	6.5	7.6	1.2 (0.7;1.6)^a^	<0.001	6.9	7.8	0.9 (0.6;1.1)^a^	<0.001
*Prenatal care (no. of visits)*												
None	4.4	1.4	-	-	5.5	3.3	-	-	8.3	3.0	-	-
≤6 or UD	32.5	17.7	-	-	40.1	27.6	-	-	35.8	33.5	-	-
≥7	63.2	80.9	-	-	54.5	69.2	-	-	55.9	63.5	-	-
LBW None	16.5	24.6	3.1 (1.4;4.8)^a^	<0.001	13.5	16.9	1.3 (−1.4;4.1)	<0.001	12.9	17.4	2.4 (0.6;4.1)^a^	<0.001
LBW ≤6 or UD	9.7	15.3	3.2 (2.4;4.0)^a^	<0.001	8.2	12.3	2.6 (2.1;3.1)^a^	<0.001	9.3	11.5	1.3 (0.2;2.4)^a^	<0.001
LBW ≥7	5.5	5.9	0.4 (−0.1;0.8)	0.011	5.9	5.7	0.3 (−0.2;0.8)	0.004	5.8	5.6	−0.1 (−0.3;0.0)	0.002
Gestational age (weeks)												
<37	5.5	8.2	-	-	3.9	9.7	-	-	5.2	9.6	-	-
≥37	94.5	91.8	-	-	96.1	90.3	-	-	94.8	90.4	-	-
LBW <37	63.1	57.4	−0.5 (−0.8;−0.2)	<0.001	62.1	44.9	0 (−1.7;1.8)	0.019	56.1	46.1	−1 (−2.3;0.3)	<0.001
LBW ≥37	4.1	3.4	−1.4 (−1.8;−0.9)^a^	<0.001	4.9	4.0	−0.7 (−2.7;1.3)	<0.001	4.9	3.9	−1.6 (−2.1;−1.1)^a^	<0.001
Mode of delivery												
Caesarean section	44.2	56.6	-	-	49.2	59.5	-	-	42.9	55.2	-	-
Vaginal delivery	55.8	43.4	-	-	50.8	40.5	-	-	57.1	44.8	-	-
Caesarean section LBW	7.3	8.4	0.5 (0.0;1.1)^a^	<0.001	6.3	8.0	1.6 (1.2;2.0)^a^	<0.001	7.1	8.0	1 (0.6;1.4)^a^	<0.001
Vaginal delivery LBW	7.5	7.1	−0.2 (−0.6;0.2)	0.078	8.1	8.0	0 (−0.4;0.5)	0.929	8.6	8.0	−0.5 (−1.1;0.2)	<0.001

^a^ *AAPC* average annual percentage of change

**Table 3:** Simple and Multivariate Poisson Regression for low birth weight year by year, and adjusted for each variables (maternal age, maternal educational level, prenatal visits, gestational age, and mode of delivery yearly), and all variables together in Brazil and its regions, according to the 26 state capitals and Brazil’s capital, Brasília, between 1996 and 2011

Year/Crude and adjusted analysis according to variables	North	Northeast	Southeast	South	Central West	Brazil
	RR (95 % CI)^a^	RR (95 % CI)^a^	RR (95 % CI)^a^	RR (95 % CI)^a^	RR (95 % CI)^a^	RR (95 % CI)^a^
Year	1.009	1.007	0.998	1.003	1.007	1.002
	(1.008;1.010)	(1.006;1.008)	(0.997;0.999)	(1.001;1.004)	(1.006;1.009)	(1.002;1.002)
Year and maternal age	1.010	1.007	0.998	1.003	1.008	1.002
	(1.009;1.012)	(1.006;1.008)	(0.997;0.998)	(1.001;1.004)	(1.006;1.009)	(1.002;1.003)
Year and educational level	1.014	1.011	1.004	1.010	1.011	1.008
	(1.012;1.015)	(1.010;1.012)	(1.003;1.005)	(1.008;1.011)	(1.010;1.012)	(1.007;1.008)
Year and prenatal care	1.011	1.009	1.013	1.018	1.014	1.010
	(1.010;1.012)	(1.008;1.010)	(1.012;1.014)	(1.016;1.019)	(1.013;1.016)	(1.010;1.011)
Year and gestational age	1.006	0.990	0.990	0.991	0.993	0.992
	(1.004;1.007)	(0.989;0.991)	(0.989;0.990)	(0.990;0.992)	(0.992;0.994)	(0.991;0.992)
Year and mode of delivery	1.010	1.009	0.998	1.002	1.008	1.003
	(1.009;1.012)	(1.008;1.01)	(0.998;0.999)	(1;1.003)	(1.007;1.009)	(1.002;1.003)
*Year and all variables*	1,005 (1,004;1,006)	0,994 (0,993;0,995)	0,996 (0,995;0,996)	0,999 (0,998;1,001)	0,994 (0,992;0,995)	0,995 (0,995;0,996)

^a^Relative Risk and 95 % confidence interval

The prevalence of LBW was evaluated year by year, grouping all state capitals and the federal capital according to their geographic region. The Average Annual Percentage of Change (AAPC) [28], used to estimate a summary measure of segment trends that best fit the data, was used to assess the possible time trend in LBW rates when considering all regions together and each region separately according to each variable. To evaluate the annual influence of the independent variables on LBW trends, an adjusted sequential Poisson regression model was used. Some authors indicate the use of Log Binomial Regression to investigate this outcome. However, as some models of Log Binomial Regression didn’t converge, the Poisson Regression was used [29]. First, a simple regression with the year as an independent variable was calculated considering the annual relative risk (RR). Then, the variables that were considered as risk factors were included one by one in this multivariate model to evaluate how each one would influence the annual RR. Finally, a third analysis was performed including all the explanatory variables in the model at once.

Database processing and analyses were performed using the SPSS software, version 18 and Jointpoint Regression Program, Version 4.2.0. The present study was approved by the Research Ethics Committee at the Hospital de Clínicas de Porto Alegre (Protocol 120323).

## Results

During the period studied, the 11,200,255 single births that occurred were included in the study. There was a decrease of 12 % in the number of single births between 1996 (757,125) and 2011 (672,217) mainly in Southeast and South regions. LBW rates showed a stability of around 8 % considering all capitals together.

There was a significant increase in the rates of LBW according to the AAPC in the less developed regions: North (1 [95 % confidence interval (CI): 0.4, 1.6]), Northeast (0.7 [95 % CI: 0.4, 1.0]), and Central West (0.7 [95 % CI: 0.4, 1.1]). In the more developed regions, Southeast and South, the rates of LBW were higher but remained fairly stable during the period (Table 1).

There was a 2 % decrease from 1996 to 2011 in the number of adolescent mothers and a 5.1 % increase of 35-year-old or older mothers in all Brazilian regions. The number of mothers with less than 8 years of schooling decreased 33.1 %. The number of mothers who did not have any antenatal visits decreased 5.3 %, whereas there was an increase of 7.6 % of mothers who had seven or more antenatal visits in all regions. There was a significant increase of 4.4 % of preterm newborns (<37 weeks). The number of women giving birth by caesarean section increased from 42.9 to 55.2 % in all regions. The highest rate (60.3 %) was reached in Northeast region where there was an increase of 22.3 % of caesarean section during the period (Table 2).

Considering maternal education for each level by the AAPC (Table 2), a significant increase of 1.5 % in LBW risk among mothers with higher education (≥12 years) could be observed each year, which was more evident in the North region (3.2 %); while among those with lower education (<8 years), no significant alterations were observed.

When considering the Multivariate Poisson Regression by including the variables one by one, the antenatal care coverage in all Brazilian regions was associated with an increase in LBW rates among mothers who did not attend antenatal care visits (2.4 % per year) and a decrease among those who attended more than seven visits (−0.1 % per year). Regarding the type of delivery, there was an increase of 1 % per year in LBW rates among newborns delivered by caesarean section in all Brazilian regions, except the Southeast (Table 3).

In the full model (Table 3), the results showed that the risk of LBW was reduced in all regions each year with all variables included in the model. This result was similar when the data was analyzed by LBW, year, and the gestational age in the adjusted model.

## Discussion

This is the largest time series study in Brazil with a greater number of individuals, and it was able to demonstrate a demographic and epidemiological transition in all Brazilian regions, represented by an intense reduction of live births, an increase in the number of late pregnancies, and changes in LBW rates in each region. This study also showed improvements in maternal education and health care, positively influencing the decrease of LBW risk in all regions, and the increase in the gestational age shows a large influence in the increase of the LBW rates.

The demographic transition has presented different stages according to the level of development of each region [30–32]. In less developed areas (the North and Northeast regions) LBW rates increased, whereas in more developed regions the higher rates were stable, probably due to an increase in the quality of the system information and data collection over the years, and higher access to the health system as a whole [22].

A previous study found that trends varied among Brazilian regions, and a higher LBW rate was seen in the more developed regions compared to less developed ones. This phenomenon, called the “low birth weight paradox,” associated the availability of health care and interventions with social conditions [19]. In the same context, the Southeast and South regions present lower rates of infant mortality [19, 33–35] and have the highest number of women in childbearing age who have private health insurance plans and effectively use these services, including the option of caesarean section [34, 36].

In Brazil, the demographic transition may also be evidenced by a decreasing number of live births, mainly in the most developed regions (Southeast and South), which is in accordance with other developed countries with higher income and lower fertility rates [37]. Despite LBW rates in Brazil and United States being similar (8 and 8.1 %, respectively), they are still considered high compared to countries such as Iceland, Finland, Sweden, Norway, and Estonia, which have less than 5 % of LBW [38, 39].

According to the World Health Organization (WHO), among the estimated 14 million pregnant adolescents aged 15–19 each year, only 10 % live in developed countries. Africa, Latin America, and the Caribbean Islands are the regions with the highest number of pregnancies in this age group [40].

The decrease in the number of adolescent pregnancy in Brazil has been notable in recent years; [25, 41] however, high rates are still a concern, mainly in regions of higher socioeconomic vulnerability [18]. In this study, adolescent mothers are an important factor for LBW, especially in the North, Northeast, and Central West regions, presenting lower socioeconomic development.

Nevertheless, pregnancy in 35-year-old or older women also influences LBW rates [42, 43]. The Southeast and South regions presented the highest LBW rates in 35-year-old or older women. These regions also showed higher maternal education level and a greater number of antenatal visits, reinforcing the “low birth weight paradox” mentioned above.

The analysis of the adjusted model investigating the influence of determinants on LBW rates one by one and under a temporal analysis suggested that maternal educational levels and antenatal visits influenced the reduction of these rates. This influence was higher in the Southeast and South regions, as they had a larger number of women with more than 8 years of schooling and greater antenatal care coverage. Maternal educational level, which is considered an important indicator of socioeconomic status, significantly increased in different regions in Brazil. The positive effect of maternal education on child health has already been described in recent years. However, this analysis took into consideration each variable as a whole, and in the descriptive analysis, 12 or more years of schooling presented an increase in LBW rates [44].

Despite the improvement in the antenatal care coverage in the country, the North and Northeast regions seemed to have fallen short of adequate levels of access to antenatal care. In these regions, there was an increased risk for LBW among infants whose mothers had six or fewer antenatal visits. It is worth mentioning that quantitative evaluation of antenatal care is quite insufficient to determine the quality of obstetric care [45]. Similar findings have also been found in other countries, such as the United States, where late antenatal care was associated with a likelihood three times higher than usual of LBW infants, mostly due to prematurity [46].

The most impactful influence of lower maternal educational level and no antenatal visits on LBW was found in the Southeast and South regions. Although great parts of the population can benefit from the advantages of urbanization and socioeconomic development promotion in these regions, current policies and health education programs are likely still not enough to reach the most vulnerable social groups.

The findings revealed an increasing number of preterm newborns in the country, especially in the Northeast, Central West, and North regions. Nevertheless, the highest number of preterm newborns and LBW rates were found in the Southeast and South regions; as these regions offer better health care for the mother and the child, the survival of preterm newborns is probably due to the accessibility of the health system [19]. Thus, improvements in obstetric, perinatal, and neonatal care, especially in these regions, contribute positively in the outcomes of high-risk pregnancies. This could serve as one of the explanations for high preterm birth rates.

In recent years, some researchers have shown evidence that preterm newborns and LBW are associated with an increased number of caesarean sections in Brazil [26, 33, 34, 41, 47]. In the Southeast and South regions, which presented a higher percentage of caesarean sections in 1996, an increased number of 35-year-old or older pregnant women was observed. The relationship between caesarean sections and negative perinatal outcomes has been investigated and some authors confirmed the association of caesarean section with LBW [41, 48, 49]. However, despite increasing caesarean sections rates, this effect was not significant in this study, probably due to an increase in LBW rates in both types of deliveries.

The number of caesarean sections in the private health system in Brazil is alarming, around 90 % of all births [50]. This suggests that there are non-clinical factors influencing the choice for one type of delivery. The improvement in diagnosing and assisting pregnant women make it possible to induce delivery and caesarean sections [51]. Recently, researchers have identified that caesarean sections are closely related to white, socioeconomically stable, adolescent mothers who have private health plans [52]. The prevalence of preterm newborns and caesarean sections seems to be a problem related to ethical issues. A study in private hospitals in Brazil suggest that changes based on evidences in the model of perinatal care may reduce the prevalence of caesarean sections and increase good antenatal practice with no adverse effects [53].

In the North and Northeast regions, there was a significant trend of increased LBW rates in vaginal deliveries. This could be related to poor social conditions and inadequate access to health care [19, 54]. Another recent study showed that the difference of LBW rates between the two modes of delivery decreased after 2006 in Brazil [55].

The state capitals of the North and Northeast regions presented a higher number of adolescent pregnant women, reduced access to antenatal care, lower maternal education, increased trend of preterm newborns, and LBW rates. Contrarily, the Southeast and South regions showed a higher number of 35-year-old or older pregnant women, higher maternal education level, and higher rates of preterm newborns and caesarean sections. Furthermore, despite the trend of stabilizing the LBW rate, considering all Brazilian regions together, the Southeast and South regions still present the highest rates when compared with other regions.

At the beginning of the period studied, the behavior of the variables obtained for the Central West region were closer to those found in the North and Northeast regions. However, the Central West region presented results similar to the Southeast and South regions in 2011. The results likely reflect a major transition in the socioeconomic conditions in this region during the period studied and its impact in the health indicators [56].

These regional differences suggest the existence of polarized social inequality. On one side, there is limited access to education and health associated with other factors, such as poor sanitation and poor living conditions, lack of adequate housing, higher unemployment, and lower wages. On the other side, where there is greater socioeconomic development, greater parts of the population have more access to various resources, including private health insurance plans and new technologies in health.

In this context, Brazil in general has improved its health indicators, due to significant advances in the sphere of social determinants (greater urbanization, universalized primary education, and economic stability, for instance) and the implementation and consolidation of the Brazilian Unified Health System (SUS) [54].

This study has some limitations. Some information, such as the quality of antenatal care received by mothers, maternal substance use (such as alcohol, drugs, or tobacco), or even the existence of gestational diseases and maternal nutrition were not available in the SINASC database until 2011. Similarly, gestational age was expressed in weekly intervals until 2011, which didn’t allow an accurate calculation for determining newborns with intrauterine growth restriction. These data are important and these factors associated with LBW could be better investigated. No linear regressions for all variables together were performed, though that was not the scope of the present study. Brazil has been able to reduce the level of poverty since 1994, enabling an ascending social mobility that influences the access to private health plans and related technologies, especially in the capitals. Yet the results may be different if the countryside is considered instead of the Brazilian capitals and Brasilia.

Over the past few decades, several nationwide public health policies have been conducted focusing on maternal and child care. Focus on reducing maternal and child mortality and their associated factors have led to the development of laws and programs to implement health services and actions. However, despite significant advances in the quality of maternal and child care in Brazil, it is necessary to focus on the regional characteristics of the country with its large territory and great geographical diversity [24, 57]. Therefore, the development of strategies for health management and care according to regional specificities should consider social inequalities and cultural differences.

Thus, these findings provide an increase of knowledge related to epidemiological and demographic transition, offering information to assess national policies of maternal and child health through the perspective of time. Such information can contribute to the development and management of actions that may be more effective if targeted to Brazilian regional peculiarities. Special policies for each age range focusing on regions of greater vulnerability could be a great asset. It is known that educational and maternal age has an important role in pregnancy as well as the emotional and social conditions of the pregnant woman. The improvement of education and antenatal care policies are important, and they can positively reflect not only the decrease of LBW with favorable antenatal and postnatal effects, but better health conditions for the whole population.
